# Effect of boiling and oven roasting on some physicochemical properties of sunflower seeds produced in Far North, Cameroon

**DOI:** 10.1002/fsn3.2637

**Published:** 2021-11-07

**Authors:** Noel Tenyang, Roger Ponka, Bernard Tiencheu, Fabrice Tonfack Djikeng, Hilaire Macaire Womeni

**Affiliations:** ^1^ Department of Biological Science Faculty of Science University of Maroua Maroua Cameroon; ^2^ Department of Agriculture Livestock and By‐Products National Advanced School of Engineering University of Maroua Maroua Cameroon; ^3^ Department of Biochemistry Faculty of Science University of Buea Buea Cameroon; ^4^ School of Agriculture and Natural Resources Catholic University Institute of Buea Buea Cameroon; ^5^ Department of Biochemistry Faculty of Science University of Dschang Dschang Cameroon

**Keywords:** lipid oxidation, lipid stability, nutrient composition, sunflower seeds

## Abstract

The effects of different processing methods on proximate composition, total phenolic content, antioxidant activity, lipid oxidation, and mineral contents of sunflower seeds produced in Far North Region of Cameroon were evaluated. Mean moisture, ash, lipid, protein, fiber, and carbohydrate contents of raw sunflower seeds were 6.60%, 2.55%, 44.65%, 20.17%, 4.08%, and 21.25%, respectively. The changes in moisture, ash (excepted in boiled samples), lipid, protein, fiber, and carbohydrate (excepted in roasted samples) were found to be significant for all cooking methods. Ash and lipid contents of samples roasted at 120°C were found to be significant when compared with other cooking methods. Antioxidant activity increased with treatment. After processing, the acid, peroxide, and thiobarbituric acid values increased significantly, whereas iodine value decreased. The roasting process improved the induction time, and samples roasted at 120°C were found to have the highest induction time (2.29 ± 0.09 hr). Raw sunflower seeds were good sources of potassium (K), magnesium (Mg), calcium (Ca), iron (Fe), zinc (Zn), and manganese (Mn). Increase in contents of Ca, Mg, Zn, Cu, and Fe was observed during processing. Roasting compared with boiling appeared to be the best cooking method of sunflower seeds concerning nutrient content, antioxidant stability, and lipid stability.

## Graphical abstract

Boiling and ovendry temperature affected the nutritional value of sunflower seeds. The protein, lipid, and ash contents increased significantly in all processed samples, whereas moisture content decreased, except in boiling samples. Roasting compared with boiling appeared to be the best cooking method of sunflower seeds concerning nutrient content, antioxidant stability, and lipid stability.

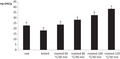

## 1 INTRODUCTION

Sunflower (*Helianthus annuus* L.), native to North America, is a plant of Asteraceae family. It was grown as an ornamental and medicinal plant until the 17^th^ century. After which, it became one of the major annual crops in the world, cultivated for edible oil. *H*. *annuus* L. can be grown successfully in arid and semi‐arid regions (Iqbal et al., 2001). According to FAO (2007), the production of sunflower seeds in the world increased from 26 to 31 million metric tons between 2004 and 2006, whereas the world production of sunflower oil was 10 million metric tons from 2005 to 2007. Russian Federation and Ukraine were the largest producers of sunflower accounting for 25% and 22% of the total world production, respectively, and in Africa, the largest sunflower seed producer was South Africa with 46.1% of total continent's production (FAO, 2007).

The importance of sunflower as source of edible oil and high‐quality protein is continuously increasing. The nutritional quality of *H*. *annuus* is comparable with soybean, and most other oilseed proteins including conventional legumes. Their potential as a dietary protein source in animal feeds is well recognized (Olvera‐Novoa et al., 2002). Protein contents of the seeds ranged from 20% to 30% (Ezra et al., 2014). More importantly, the proteins of sunflower contain tryptophan, which is an important amino acid essential for growth, especially in children. Sunflower seeds contain about 48%–53% edible oil, and this oil is used for a variety of cooking purposes. Due to their light yellow color, good flavor, high linoleic acid content, and high smoke point, sunflower oil is considered premium to other vegetable oils (Sharma et al., 2013). Research studies have shown that oleic and linoleic acids present in sunflower oil help to reduce the low density lipoprotein cholesterol and total cholesterol, decreasing the chance of coronary artery diseases (Chowdhury et al., 2007). Sunflower seeds are also a rich source of carbohydrate, ash, dietary fiber, vitamin E, B vitamin complex (especially vitamin B1 and B5), and minerals. Additionally, they are a good source of antioxidants, such as flavonoids and phenolic acids (Pasko et al., 2009).

Nadeem et al. (2010) reported that the chemical composition of *H*. *annuus* seeds varies and depends on climatic conditions, genetics, varieties, soil type, and processing methods.

In Africa, seeds are mostly used in human nutrition and animal feeding. Before they have been used, they are submitted to different processing methods. The most popular methods of preparing sunflower seeds are boiling and roasting. The main goal of technological treatment is to improve the organoleptic characteristics, the antioxidant activity, and bioactive molecules and to enhance the nutritional quality of seeds. However, processing will also cause chemical changes that may negatively affect the nutritional value of seeds. Lipid oxidation, for example, strongly affects shelf life of oil. Oxidation reactions depend on many factors such as amount of unsaturated fatty acids in oil, enzymatic activity, and presence of natural antioxidants (Ozdenir & Devres, 2000).

Several studies have reported the effects of processing of sunflower seeds in some countries; these include chemical, nutritional, and antinutritional study of new varieties of oil seeds from sunflower, safflower, and groundnut (Satish & Shrivastava, 2011); effect of some processing techniques on the proximate and antinutrient composition of *H*. *annuus* seeds (Sharma et al., 2013); effect of different air‐drying temperature on sunflower seed oil and ash content (Matin et al., 2017); and effect of processing on the proximate composition of sunflower seeds (Adesina, 2018).

Though considerable attention had been given to the study of sunflower seeds, there is however very limited information on chemical composition and the effects of processing on *H*. *annuus* grown in Cameroon. We can hypothesize that nutritional value of sunflower seeds from different locations is different, and boiling and ovendrying temperature affect differently the physicochemical properties of sunflower seeds cultivated in Far North, Cameroon.

Therefore, the aim of the present work was to investigate the effect of boiling and ovendrying temperature on proximate composition, phenolic content, lipid oxidation, and mineral contents of sunflower seeds cultivated in Far North Region of Cameroon.

## 2 MATERIALS AND METHODS

### 2.1 Plant material and study site

The sunflower seeds used in the present study are commonly cultivated in Maroua (Far North Region of Cameroon). This area is located between latitude 10° and 13° North and between longitude 13° and 16° East. It has a tropical Sudano‐Sahelian climate type with a long dry season of about 8 months and a short rainy season of 4 months (Tenyang et al., 2017). Four kilograms of dry raw sunflower seeds (Trt‐9 variety) was collected from the Institute of Agricultural Research for Development (IRAD) Maroua in March 2018 and transported in polyethylene sacks to Food Biochemistry Laboratory. In the IRAD, after harvesting and threshing, the seeds were dried in the shade for 20 days at mean room temperature of 30°C. After drying, only seeds that were not damaged were chosen for further analyses.

### 2.2 Chemicals reagents

Folin–Ciocalteu reagent, gallic acid, bicarbonate of sodium, starch, hydrocloric acid 35%, and sulfuric acid 98% were purchased from *SD* Fine Chemicals Limited. Acetic acid, thiobarbituric acid (TBA), carbon tetrachloride, sodium thiosulfate, ethanol 95%, Wijs reactif, and phenolphthalein were procured from HiMedia Laboratories Pvt Ltd. Hexane and standard solutions for atomic absorption spectrometry (Ca, Mg, Na, K, Fe, Zn, Mn, and Cu; purity 99.99%) were purchased from Courtage Analyses Services (Mont‐Saint‐Aignan, France). All reagents used in the analysis were of analytical grade.

### 2.3 Heat treatments

The dried sunflower seeds were divided into three different groups. The first group (200 g) was considered as control, the second group (800 g) was divided into four subgroups of 200 g each for roasting processing (60, 80, 100, and 120°C for 30 min each), and the third group (200 g) was used for boiling treatment.

For the roasting process, 800 g of whole sunflower seeds was arranged in a single layer in aluminum foil dishes (12.8 cm) and then placed in electric air‐drying oven model G48 (Balay) on a stainless steel grill with the thermostat. The seeds were roasted at 60, 80, 100, and 120°C for 30 min after the set temperature was attained (60, 80, 100, and 120°C, respectively).

For the boiling process, 200 g of whole seeds was dipped into boiling water at ~97°C at ratio of 1:5 seed : water for 30 min.

The raw, roasted, and boiled sunflower seeds were allowed to cool down to ambient temperature and then grounded in an electric grinder (Panasonic), and the obtained powder was stored at 4°C for further analysis.

### 2.4 Lipid extraction

Seventy grams of sunflower flour was soaked in 300 ml of hexane during 72 hr with shaking from time to time at room temperature (~25°C). The mixture was filtered on Whatman paper no. 4, and hexane was concentrated under vacuum using rotary vapour (40°C). After which, the supernatant was collected and evaporated to obtain the solvent‐free oil. Then, the extracted oil was dried using anhydrous sodium sulfate.

### 2.5 Extraction of sunflower polyphenols

The extraction of sunflower was performed following the method described by Womeni et al. (2016). Twenty grams of each processed sunflower powder was extracted with 400 ml of methanol for 48 hr at room temperature, with regular shaking during extraction. The extract was filtered with Whatman filter paper no. 1, and the residue was again extracted with 200 ml of methanol to ensure maximum extraction of phenol compounds. The combined filtrates were evaporated at 40°C under reduced pressure for removal of the solvent. The dried extract was used for further analysis.

### 2.6 Analytical methods

#### 2.6.1 Proximate composition of sunflower seeds

Moisture, lipid, ash, protein, and crude fiber in the samples were determined using standard analytical methods described by AOAC (1990) procedures. Moisture content was determined by drying sunflower seeds in oven at 103°C until a constant weight was achieved according to the AOAC procedures 925.40. Ash content was determined by burning sunflower seeds at 550°C according to the AOAC procedures 942.05. Nitrogen (N) contents were determined using micro‐Kjeldahl method, according to AOAC procedures 984.13; the N content was multiplied with 6.25 to estimate the crude protein of these samples. Lipid content was determined using Soxhlet apparatus with hexane, following AOAC 963.15 methodology. Crude fiber was determined using a fiber digester according to the AOAC procedures 973.18. The carbohydrate content was calculated by difference. All samples were analyzed in triplicates.

#### 2.6.2 Total polyphenol of sunflower seeds

The total phenolic content (TPC) of sunflower seed samples was determined using the Folin–Ciocalteu colorimetric method, as described by Gao et al. (2000). The seeds extracts (20 µl) were mixed in a test tube with 0.2 ml of Folin–Ciocalteu reagent and 2 ml of distilled water and incubated at room temperature for 3 min. Following this, 1 ml of 20% solution of Na_2_CO_3_ was added to the mixture and re‐incubated for 24 hr at room temperature. The absorbance of the resulting blue color was measured using a quartz cuvette at 765 nm. The procedure was repeated to all standard gallic acid solutions, and standard curve was obtained. The content of phenols in the test samples was determined from the standard curve, and the results were expressed as milligrams gallic acid equivalents per gram of extract.

#### 2.6.3 Antioxidant activity of sunflower seeds

The method described by Braca et al. (2002) was used to determine the ability of each extract to scavenge the DPPH radical. Synthetic antioxidant, butylated hydroxytoluene (BHT), which is a recognized powerful radical scavenger, was used as positive control. The antioxidant activity (AA) was calculated according to the formula:

AA% = [(Abs_control_ − Abs_sample_) × 100/Abs_control_].

#### 2.6.4 Chemical analysis of sunflower oil

The determination of free fatty acid (FFA) content and peroxide value (PV) of sunflower seed oils was performed according to the procedure of AFNOR (1981). Its iodine and TBA values were evaluated as described respectively by O'Keefe and Pike (2010) and Draper and Hadley (1990).

The resistance of sunflower oil to auto‐oxidation expressed as induction period in hours was determined using an automated Rancimat model 743 (Metrohm). About 5 g of each oil sample was weighed in individual reaction vessels of the instrument, and vessels were placed in a heating block for 10 min for preheating of sample. After that, air was supplied by a built‐in pump at flow rate of 20 L/h. Temperature was adjusted to 110°C, and absorption vessels filled with 60 ml deionized water were connected with reaction vessels via Teflon tubing. The induction period was automatically recorded with changes in water conductivity (Yanishlieva‐Maslarova & Heinonen, 2001).

#### 2.6.5 Mineral composition of sunflower seeds

For the determination of minerals, sunflower seeds were ashed at 550°C, and the ash was boiled with 10 ml of 20% HCl in a beaker and then filtered into a 100 mL standard flask to determine the mineral content. Calcium (Ca), magnesium (Mg), sodium (Na), potassium (K), iron (Fe), zinc (Zn), and copper (Cu) were determined by atomic absorption spectrometer (Varian 220FS SpectrAA). Reference sample from parts of the daily routine in the laboratory was used for quality control. Certified reference material 1570a was purchased from the National Institute of Standards and Technology. After initial standardization of techniques during a pilot study, the samples were treated identically. Mineral contents of the samples were determined from calibration curves of standard minerals. All samples were analyzed in triplicate.

#### 2.6.6 Statistical analysis

Data were analyzed by one‐way analysis of variance using Statistical Package for the Social Sciences (SPSS version 16.0). Comparison was made between species and treatments. Significance level was set at *p* < .05.

## 3 RESULTS AND DISCUSSION

### 3.1 Effect of processing methods on proximate composition of sunflower seeds

The effect of common processing methods on proximate composition of sunflower seeds is presented in Table 1. The moisture content of samples was 6.60% for raw sample, 7.10% for boiled sample, and 4.4%, 3.38%, 2.76%, and 2.32%, respectively, for roasted samples at 60, 80, 100, and 120°C for 30 min. The raw sample was significantly (*p* <.05) higher in moisture content when compared with processed samples. The roasted sample at 120°C for 30 min had the lowest moisture content. Boiling process was found to slightly increase the moisture content of boiled sunflowers seeds. Increase of the moisture of boiled seeds may be due to the absorption of water by seeds during processing. During roasting process, decrease of moisture content may be linked to dehydration due to the increase of temperature in dry roasting conditions. These findings in the present study are similar to the results reported by Tenyang et al. (2017) when analyzing the effect of boiling and roasting on proximate composition of two sesame varieties consumed in Far North, Cameroon. Some investigations have shown that low moisture content of food is a desirable phenomenon, because the microbial activity is reduced. So as low moisture content in food samples increased the storage time of food product, this indicates that roasted sunflowers seeds and particularly those roasted at 120°C for 30 min can be stored for a long time and will be less prone to microbial attack during storage (Oyenga, 2013).

**TABLE 1:** Effect of processing methods on chemical composition (%) of sunflower seeds

Samples	Moisture	Ash	Lipid	Proteins	Fiber	Carbohydrate
Raw	6.60 ± 0.05^b^	2.55 ± 0.08^e^	44.65 ± 0.05^e^	20.17 ± 0.00^e^	4.08 ± 0.04^b^	21.25 ± 0.49^b^
Boiled	7.10 ± 0.10^a^	2.68 ± 0.01^de^	46.95 ± 0.02^d^	17.33 ± 0.01^f^	3.02 ± 0.07^d^	23.07 ± 0.22^a^
Roasted 60°C/30 min	4.44 ± 0.01^c^	2.83 ± 0.04^d^	47.12 ± 0.09^d^	21.16 ± 0.03^bd^	4.43 ± 0.00^b^	20.01 ± 0.30^b^
Roasted 80°C/30 min	3.38 ± 0.00^d^	3.30 ± 0.13^c^	49.16 ± 0.10^c^	21.83 ± 0.01^c^	4.76 ± 0.01^a^	17.18 ± 0.18^c^
Roasted 100°C/30 min	2.76 ± 0.03^e^	3.55 ± 0.01^b^	52.21 ± 0.08^b^	23.17 ± 0.05^a^	4.94 ± 0.10^a^	15.45 ± 0.67 cd
Roasted 120°C/30 min	2.32 ± 0.03^f^	3.95 ± 0.03^a^	55.28 ± 0.02^a^	22.07 ± 0.02^b^	4.04 ± 0.16^c^	14.42 ± 0.08^d^

Note

Values are means ± standard deviation (*n* = 3). Mean values in the same column with different superscript letters are significantly different (*p* < .05).

The ash content of raw sunflower seeds was 2.55%, which is low compared with the value reported by Adesina (2018) in the study of the proximate composition of sunflower seeds grown in Nigeria. Variation may be linked to species or geographical environment. It was observed that boiling process has no significant effect (*p* > .05) on ash content of sunflower seeds. However, seeds roasted at 60°C for 30 min have very close ash contents to boiled samples, whereas those roasted at more than 60°C (80, 100, and 120°C) led to an increase in ash content, and roasted samples at 120°C were found to have the highest ash content. These results are different from the findings of Tenyang et al. (2017). However, they are in agreement with the finding of Matin et al. (2017) who noted that increase of dry temperature between 60 and 100°C increases the ash content of sunflower seeds hybrid PR63D82. The increase in ash content noted in roasted samples in this study is explained by the reduction in moisture content as presented in Table 1.

The results show that the lipid contents of sunflower seeds ranged between 44.65% and 55.28%, with significant change in content between each treatment. A roasted sunflower seed at 120°C for 30 min was found to have the highest lipid content, whereas the raw sample was found to have the lowest lipid content. The amount of lipids in raw sample was higher to that reported by Satish and Shrivastava (2011) in India. The observed variation may be linked to species, the climatic condition, and the type of oil. Increase of lipid content during roasting may be linked to decrease of moisture during processing, and variation in lipid content of different samples may be due to the processing method applied. These results are in accordance with the finding of Matin et al. (2017) but are different from the finding of Djikeng et al. (2018) who reported that roasting process rather decreases significantly the lipid content of fermented cocoa beans. The high content of lipid makes sunflower seeds a potential for the oil industry and culinary applications.

Raw sunflower seeds were rich in protein (20.17%). This value was higher than those reported by Muesser et al. (2017), who obtained 10.91% of sunflower seeds cultivated in Turkey, but still lower than that noted by Ezra et al. (2014; 29.6%) in Tanzania. Protein content of sunflower seeds decreased with boiling treatment, whereas it increased with roasting temperature, and roasted sample at 100°C for 30 min was found to have higher protein content (23.17%). At 120°C, the protein content of roasted sunflower seeds decreases significantly (*p* < .05) compared with roasted samples at 100°C for 30 min. A decrease in protein content during boiling treatment may be due to their migration into cooking water during processing. Fagbemi (2007) had also reported that decreases of protein content in boiled soybean may be attributed to leaching during boiling process. However, their increase during roasting process may be linked to dehydration that occurred. The decrease in protein content at 120°C observed in this study is not in line with the finding of Tenyang et al. (2017) in roasted sesame seeds at the same conditions.

In this study, the results obtained also indicated significant variation (*p* < .05) on the crude fiber between raw, boiled, and roasted samples. The crude fiber of raw sunflower seeds (4.08%) was lower than 23%, values reported by Ezra et al. (2014) in Tanzania sunflower seeds. The observed difference may be linked to species and geographic environment. The crude fiber content of sunflower seeds decreased with boiling process but increased with roasting temperature, and roasted sample at 120°C for 30 min contained relatively high amount of fiber. These observations are in accordance with the finding of Tenyang et al. (2017) in roasted sesame seeds.

As shown in Table 1, the carbohydrate content of raw sunflower seeds was 21.25% and still lower than those noted by Satish and Shrivastava (2011) in India. During boiling treatment, a significant increase (*p* < .05) in carbohydrate content was observed. Compared with raw samples, roasting processing decreased the total carbohydrate content, and roasted samples at 120°C at 30 min were found to have the lower carbohydrate content (14.42%). Onyeike et al. (2015) also reported the decrease of total carbohydrate content during roasting process of walnut seeds. These may be linked to the Maillard reaction occurring during processing, in which some carbohydrates reach with other compounds like amino acids to form sensory compounds, improving the taste, color, and odor of processed foods.

### 3.2 Effect of processing methods on the phenolic content and antioxidant activities of sunflower seeds

#### 3.2.1 Total phenolic content

**FIGURE 1:**
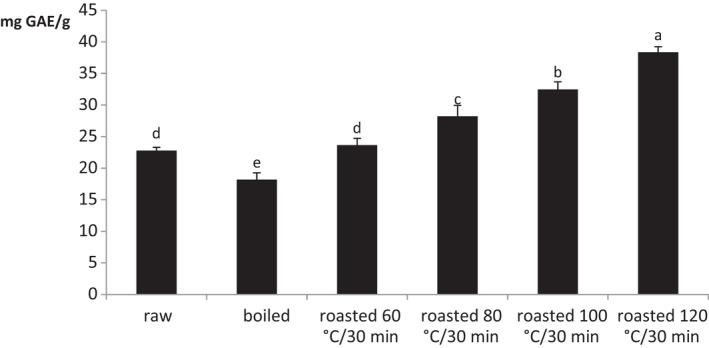
Changes in total phenolic content of sunflower seeds during processing

The phenolic compounds present in fruits and seeds are the main antioxidants protecting the body from reactive oxygen species that cause cell damage. Figure 1 shows the TPC of raw, boiled, and roasted sunflower seeds. As seen in the figure, the TPC of raw sample was 2279 mg GAE/100 g. This result was slightly lower than the value obtained by Nadeem et al. (2010) in sunflower seeds in Pakistan (2965.90 mg GAE/100 g). The TPC of sunflower seeds is closely related to the analytical methodologies, the difference in sample material and origin. During boiling process, a significant decrease was found in TPC of sunflower seeds (*p* < .05). The loss of TPC observed during this process could be due to leaching out and denaturation of polyphenols (Sun et al., 2012). There was no significant difference (*p* > .05) in TPC of the roasted sunflower seeds at 60°C for 30 min when compared with raw sample. But at 80°C and more, the TPC of sunflower increased significantly (*p* < .05), and the highest content was found in samples roasted at 120°C for 30 min. These changes were similar to those found by Behrooz et al. (2013) in roasted sesame seeds. Increases of TPC during roasting processes have been attributed to dehydration of food matrix and an improved extractability of phenolic compound from the seeds. Dewanto et al. (2002) also reported that increase in TPC in soybean during roasting treatment was primarily due to the increased release of phytochemicals as phenolic acids. The heat disrupts the cell membranes and cell walls, which releases the soluble phenolic contents from the insoluble ester bonds. The roasting temperature after 60°C is able to destroy the polyphenol oxidase enzymes, leading to the inhibition of polyphenolics degradation. Ozdemir et al. (2001) attributed the increase of TPC during roasting of hazelnuts to the increased generation of Maillard reaction product during processing.

#### 3.2.2 Antioxidant activity of sunflower seeds

**FIGURE 2:**
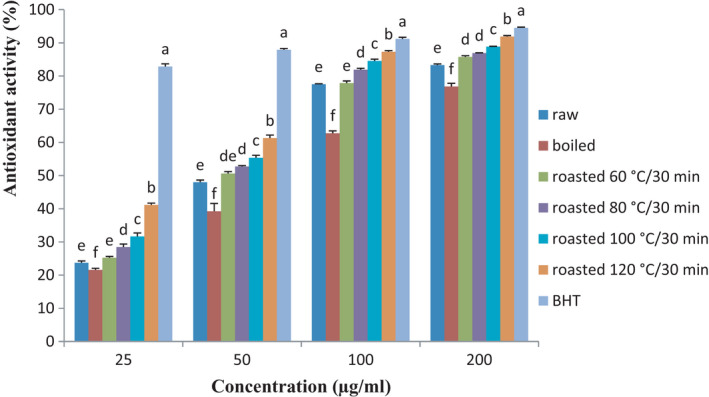
Changes in DPPH radical scavenging activity of sunflower of sunflower seeds during processing. Abbreviation: BHT, Butylated hydroxytoluene

Figure 2 presents the radical scavenging activity of raw and processed sunflower seed extract compared with synthetic antioxidant, namely, BHT. It can be seen that the activity of raw sunflower seed extracts was similar to that of the BHT at all concentration. However, the activity of all treated and untreated samples was significantly lower (*p* < .05) compared with that of BHT at all concentration. Globally, the activity of the sunflower seed extracts was significantly enhanced (*p* < .05) with their concentration and roasting temperature. Roasting treatment compared with boiling treatment significantly increased the antioxidant activity of sunflower seeds, and the sample roasted at 120°C for 30 min exhibited the highest activity. As presented in Figure 2, boiling samples showed the lowest antioxidant activity compared with untreated samples at all concentration. These results are in good agreement with those presented by Jeong et al. (2004), where it was demonstrated that roasting treatment enhances the antioxidant activity of sesame seeds. The same trend was noted by Jannat et al. (2010) when studying the effect of roasting temperature and time on TPC and antioxidant activity of sesame seeds. The variation in antioxidant activity of all samples could be attributed to the type and concentration in phenolic compound and other phytochemical constituents that occurred in each sample during processing. Djikeng et al. (2018) showed that plant extracts having high concentration in phenolic content were also exhibiting the high antioxidant activity. Consumption of sunflower seeds especially those roasted at 120°C for 30 min with high antioxidant activity may help in living organism to prevent several diseases such as cancer.

### 3.3 Effect of processing methods on sunflower oil qualities

#### 3.3.1 Acid value

Phospholipids, triglycerides, and diglycerides present in oils and fats are prone to thermal hydrolysis particularly in the presence of water, releasing fatty acids from their ester linkage, and increasing FFA. FFA content is therefore frequently used to evaluate hydrolysis extension, a very important quality issue of oils and fats. The FFA values of raw, boiled, and roasted sunflower seeds are shown in Table 2. The FFA of sunflower oil samples was in the range of 0.28%–1.65% oleic acid. The FFA of raw sunflower seed oil obtained in this study is higher than 0.10% oleic acid obtained by Issa et al. (2017) in Turkey in sunflower seed oil. However, this value is still lower than 2.35% oleic acid, value reported by Ezra et al. (2014) for sunflower seed oil in Tanzania. Variation in FFA in raw samples may be linked to the variety and the origin of the sample. FFA of sunflower seeds cooked by boiling treatment showed low value compared with untreated sunflower seeds but significantly increasing (*p* < .05) during roasting treatment, and the sample roasted at 100°C for 30 min was found to have the higher FFA content. There was no significant difference (*p* > .05) between the FFA of raw and roasted sample at 120°C for 30 min. Increase in FFA during roasting treatment is in line with the finding of Khalid et al. (2017). The increase in FFA during processing may be attributed to heat, which is responsible for lipid hydrolysis and release of FFA. Decrease of FFA during boiling may be due to their destruction in other oxidative components during processing. There is an inversely proportional relationship between the edibility of oil and the total amount of FFA. When oil has a low amount of FFA, there is a sign that the degree of their edibility is high, and the storage life is also long. The value of FFA obtained in all the processed sunflower seeds in this study revealed that all the oil samples maintained good qualities as their value did not exceed 2% oleic acid, the maximum limit of oil according to Codex Alimentarius (2013).

**TABLE 2:** Changes in acid, iodine, peroxide, and TBars values and induction time of sunflower seed oil during processing

Samples	Acid value (% oleic acid)	Iodine value (g I_2_/100 g of oil)	Peroxide value (meq O_2_/kg of oil)	TBars value (mg MDA/kg of oil)	Induction time (h)
Raw	0.56 ± 0.08^d^	102.06 ± 0.68^a^	4.48 ± 0.17^d^	0.69 ± 0.02^d^	0.75 ± 0.02^c^
Boiled	0.28 ± 0.10^e^	90.27 ± 0.09^e^	13.65 ± 0.50^a^	2.19 ± 0.18^a^	0.66 ± 0.01^d^
Roasted 60°C/30 min	0.93 ± 0.02^c^	97.65 ± 0.22^bc^	6.73 ± 0.40^c^	1.02 ± 0.06^c^	0.91 ± 0.04^bc^
Roasted 80°C/30 min	1.33 ± 0.05^b^	97.21 ± 0.22^c^	8.50 ± 0.11^b^	1.44 ± 0.00^b^	1.00 ± 0.01^b^
Roasted 100°C/30 min	1.65 ± 0.03^a^	98.35 ± 0.15^b^	9.38 ± 0.01^b^	1.62 ± 0.04^b^	1.02 ± 0.03^b^
Roasted 120°C/30 min	0.69 ± 0.02^d^	95.97 ± 0.02^d^	5.06 ± 0.85^d^	1.48 ± 0.05^b^	2.29 ± 0.09^a^

Note

Values are means ± standard deviation (*n* = 3). Mean values in the same column with different superscript letters are significantly different (*p* < .05).

Abbreviation: TBARS, thiobarbituric acid reactive substances.

#### 3.3.2 Iodine value

Iodine value (IV) is a parameter frequently used to measure the degree of unsaturation in oil. It determines their stability to oxidation after processing. Table 2 shows the IV of raw, boiled, and roasted sunflower seeds. It can be observed that IV of sunflower seed oil ranged between 90.27 and 102.06 g I_2_/100 g oil. The values of IV noted in our sample are lower than 122 and 144 g I_2_/100 g oil obtained respectively by Khalid et al. (2017) in China and Ezra et al. (2014) in Tanzania in raw sunflower seed oils. Variation may be linked to species and variety and their environment. The IV decreased significantly during processing, and the lower value (90.27 g I_2_/100 g oil) was found in oil from sunflower seeds boiled for 30 min. There was no significant variation between IV of roasted sunflower seed oil at 60°C and that roasted at 80°C. The oil of roasted sunflower seeds at 120°C compared with other roasted sunflower seeds was found to have a lower IV. Decreases in IV noted in roasted samples in this study are in agreement with the finding of Khalid et al. (2017). A decrease in IV is proportional to the reduction of unsaturated fatty acid present in oil. These characterize the oxidative changes in oils during processing. In fact, during processing, free radicals attack the double bonds of unsaturated fatty acids in sunflower seed oils, resulting in the reduce of IV of oil. Orthoefer et al. (1996) in their finding reported that heat treatment can cause the oxidative rancidity of oils and fats.

#### 3.3.3 Peroxide value

Peroxide value reflects the content of primary oxidation products that are formed during both initial and propagation phases of lipid oxidation. It is an important parameter used to assess the quality of lipid. In the present study, the PV of all treated sunflower seeds varied with process as shown in Table 2. Except roasted sample at 120°C for 30 min, there was noticeable increase in PV when the roasting temperatures increased. Boiled sunflower seed oil compared with all samples was found to present the high PV (13.65 meq/kg oil). The lowest PV (4.48 meq/kg oil) was recorded in raw sample. No significant difference (*p* > .05) was observed between the PV of raw sample and that of roasted sample at 120°C for 30 min. The increase of PV during processing indicates that exposition of sunflower seeds in water and at high temperature potentiates the formation of lipid peroxide molecules, as results of free radical attacking the unsaturated fatty acids (Nkpa et al., 1990). These results are in line with those noted by Khalid et al. (2017) who showed that the PV of oils extracted from sunflower seeds was increasing with the roasting time. A low level of PV in roasted sunflower seeds at 120°C compared with other treated samples may be due to their high level of TPC as presented in Figure 1. Apart from boiled sunflower seed oil that exhibited a PV higher than the recommended value, which is 10 meq O_2_/kg oil (FAO/WHO, 2009), all samples presented a PV lower than 10 meq O_2_/kg oil. The PVs of oil of raw and roasted samples at 120°C for 30 min were found around 5 meq O_2_/kg oil, which indicates the relatively good quality of these oils.

#### 3.3.4 TBA value

To assess the secondary oxidation state of oils or fats, the TBA test is widely used. The secondary products occur in oils and fat when hydroperoxides are decomposed into nonvolatile carbonyls during the lipid oxidation. The change in TBA of sunflower seed oil samples is presented in Table 2. A general increase in TBA value was observed when processing change. Boiled sample, which has previously presented the high PV, has also showed highest TBA value (2.19 mg MDA/kg of oil). No significant differences (*p* > .05) were noted between roasted samples at 80, 100, and 120°C for 30 min. Increase in TBA value during processing is linked to the formation of malonaldehydes obtained from the decomposition of hydroperoxides under the effect of heat. Raw sample exhibited lowest TBA value (0.69 mg MDA/kg of oil). Increase in TBA value is in agreement with the observations of Tenyang et al. (2017) who noted that the secondary product in sesame seeds increases with roasting temperature and time. The lowest TBA value in roasted sample at 60°C for 30 min means that it contains a lower secondary product oxidation compared with all the treated samples.

#### 3.3.5 Racimat test

Oxidative stability is known as the resistance to oxidation processes established through well‐defined conditions. The thermal oxidative stabilities of oils extracted from raw, boiled, and roasted sunflower seeds were evaluated using the Racimat method. This is the most common test used to assess the oxidative stability of edible oils, which is expressed as the oxidative induction time (Farhoosh & Kafrani, 2011). Induction time refers to a period of time before the chain reaction of oil oxidation begins to accelerate, and thus, a longer induction time indicates superior oxidative stability. Induction time of raw, boiled, and roasted sunflower seed oils is presented in Table 3. Raw sunflower seed oil exhibited a 0.75 hr induction time. A significant decrease (*p* < .05) in induction time was noted after 30 min of sunflower seed boiling. However, during roasting, significant increase (*p* < .05) was observed and when roasting temperature increases. The roasted sunflower seed oil at 120°C exhibited the highest induction time (2.29 hr). No significant variation was noted between the roasted sample at 60, 80, and 100°C for 30 min. Increase of induction time during roasting is in line with the finding of Velickovska et al. (2015) who noted the increase in induction time in sesame seeds oil submitted to roasting treatment. The high induction time of oil extracted from roasted sunflower seeds at 120°C suggested that they have higher oxidative stability. This high stability may be due to the high TPC of this sample and its high antioxidant activity. The short induction time in boiled sunflower seed oil indicates the decrease stability of these oils. Decrease in induction time coincides with low total phenolic acid content in boiled sample.

**TABLE 3:** Effect of boiling and roasting temperature on mineral contents (mg/100 g) of sunflower seeds

Samples	Ca	Mg	K	Na	Zn	Cu	Mn	Fe
Raw	2.82 ± 2.26^e^	314.31 ± 1.57^d^	1001.59 ± 2.00^b^	4.75 ± 0.01^c^	7.37 ± 0.03^c^	2.71 ± 0.01^d^	7.15 ± 0.01^c^	10.62 ± 0.17^d^
Boiled	2.87 ± 0.42^d^	328.56 ± 2.20^bc^	980.97 ± 1.13^c^	3.63 ± 0.01^d^	8.74 ± 0.38^b^	3.06 ± 0.01^a^	8.64 ± 0.20^b^	11.02 ± 0.04 cd
Roasted 60°C/30 min	2.91 ± 0.44^c^	325.72 ± 0.84^c^	952.55 ± 3.61^e^	4.85 ± 0.07^c^	7.81 ± 0.12^c^	2.76 ± 0.00 cd	6.32 ± 0.07^d^	10.49 ± 0.01^d^
Roasted 80°C/30 min	2.93 ± 2.59^c^	329.51 ± 0.72^bc^	960.67 ± 0.95^d^	4.93 ± 0.78^c^	7.75 ± 0.20^c^	2.78 ± 0.03^c^	7.67 ± 0.32^c^	11.92 ± 0.11^c^
Roasted 100°C/30 min	310.84 ± 0.27^b^	331 ± 0.00^b^	977.30 ± 3.81^c^	7.70 ± 0.04^b^	8.86 ± 0.04^b^	2.87 ± 0.04^b^	10.15 ± 0.24^a^	13.12 ± 0.50^b^
Roasted 120°C/30 min	323.18 ± 1.31^a^	353.62 ± 3.36^a^	1116.59 ± 2.18^a^	11.10 ± 0.00^a^	10.02 ± 0.04^a^	3.08 ± 0.01^a^	5.43 ± 0.61^e^	14.80 ± 1.07^a^

Note

Values are means ± standard deviation (*n* = 3). Mean values in the same column with different superscript letters are significantly different (*p* < .05).

### 3.4 Effect of boiling and roasting temperature on minerals content of sunflower seeds

Table 3 depicts the mineral composition of raw, boiled, and roasted sunflower seeds. As shown in this table, in sunflower seeds, macrominerals were the most important, with the highest being potassium (K), followed by magnesium (Mg), calcium (Ca), and sodium (Na). The other minerals were in lower concentrations. The K, Ca, and Na contents of sunflower seeds in this study were higher than the values reported by Bolanos et al. (2019) in Argentina sunflower seeds. However, the Mg content obtained by the same authors was higher than the value determined in this study. Concerning microminerals, iron (Fe) was the most abundant (10.62 mg/100 g), followed by zinc (Zn: 7.37 mg/100 g) and manganese (Mn: 7.15 mg/100 g). Copper (Cu) was the lowest micromineral (2.71 mg/100 g). The Fe and Mn contents in this study are highest than those obtained in some sunflower hybrid seeds in Pakistan. However, the Zn content noted by these authors is higher than 7.37 mg/100 g, value obtained in this study. The difference noted may be due to species, the different environments, and analytical techniques used. Between raw, boiled, and roasted sunflower seeds, there was a significant difference (*p* < .05) in mineral contents. The K content of all the processed samples significantly (*p* < .05) increased, and the sample roasted at 120°C for 30 min exhibited the highest K content. Boiled sample compared with all roasted samples had the lower K content. All treatments also increased significantly (*p* < .05) the Ca, Mg, and Na contents of sunflower seeds, and the highest values were found in the samples roasted at 120°C for 30 min. There was no significant (*p* ˃ .05) difference between Ca and Mg contents of roasted sunflower seeds at 60 and 80°C. Boiled sunflower seeds compared with raw sample significantly decreased their Na content. The K proportion in all processed samples was the highest, followed by Mg, Ca, and Na. The reduction of Na in the case of boiling treatment may be linked to their leaching into boiling water. Increase in all the macromineral proportion in this study after roasting treatment may be justified by the increase in ash content noted in Table 1. The Mn proportion of raw sunflower seeds was found to be 7.15 mg/100 g. This value increases significantly (*p* < .05) after boiling, roasted at 60, 80, and 100°C for 30 min. After roasting at 120°C, the Mn content of sunflower seeds decreases significantly. The results also revealed that the highest Fe, Zn, and Cu contents after processing were found in roasted sunflower seeds at 120°C. There were no significant (*p* < .05) differences between the Cu and Zn contents of roasted sunflower seeds at 60 and 80°C for 30 min. Increase of mineral contents is the consequence of heat. Umoren et al. (2005) reported that high temperature increased the digestibility of seeds that initiated the release of some minerals. Seena et al. (2006) reported that Fe present in sunflower seeds is an essential mineral in human health and plays a role in immune function, cardiovascular health, and cognitive development. Tenyang et al. (2017) also mentioned that Mn and Zn abundant in processed sunflower seeds have vital role in bone mineralization, enzyme synthesis, taste, appetite, and growth. Consumption of processed sunflower seeds may help to prevent many disorders due to mineral deficiency in humans.

## 4 CONCLUSION

Sunflower seeds cultivated in the Far North Region of Cameroon due to their high content in lipid, protein, and minerals were considered having an important nutritional value. Their proximate composition, TPC, and antioxidant activity increased considerably after boiling and roasting. The major mineral contents also increased significantly, and the highest values were found in samples roasted at 120°C for 30 min. The oil analysis showed that all processing significantly affected the quality indexes: the acid value, PV, and TBA value significantly increased during processing. However, IV considerably decreased. Boiling process was found to be the most severe treatment, negatively affecting the index qualities. The roasting process improved the induction time of sunflower seeds, and samples roasted at 120°C were found to have the highest induction time. The processed sunflower seeds cultivated in this region of Cameroon can be adopted as supplement in human and animal diet due their high quality.

## CONFLICT OF INTEREST

There are no conflicts of interest to declare.

## AUTHOR CONTRIBUTIONS

**Noel TENYANG:** Conceptualization (equal); Formal analysis (equal); Methodology (equal); Supervision (equal); Writing‐original draft (equal). **Roger Ponka:** Formal analysis (equal). **Bernard Tiencheu:** Writing‐original draft (equal). **TONFACK DJIKENG Fabrice:** Methodology (equal). **Hilaire Macaire WOMENI:** Supervision (equal); Writing‐original draft (equal).

## ETHICAL APPROVAL

This article does not contain any studies with participants or animals requiring ethical approval.

## Data Availability

Data are available upon request from the authors.
